# Spatially fractionated radiotherapy (GRID) using helical tomotherapy

**DOI:** 10.1120/jacmp.v17i1.5934

**Published:** 2016-01-08

**Authors:** Xin Zhang, Jose Penagaricano, Yulong Yan, Xiaoying Liang, Steven Morrill, Robert J. Griffin, Peter Corry, Vaneerat Ratanatharathorn

**Affiliations:** ^1^ Radiation Oncology Department University of Arkansas for Medical Science Little Rock AR; ^2^ Department of Radiation Oncology University of Texas Southwestern Medical Center Dallas TX USA

**Keywords:** GRID, helical tomotherapy, treatment planning system, dosimetric comparison

## Abstract

Spatially fractionated radiotherapy (GRID) was designed to treat large tumors while sparing skin, and it is usually delivered with a linear accelerator using a commercially available block or multileaf collimator (LINAC‐GRID). For deep‐seated (skin to tumor distance (>8 cm)) tumors, it is always a challenge to achieve adequate tumor dose coverage. A novel method to perform GRID treatment using helical tomotherapy (HT‐GRID) was developed at our institution. Our approach allows treating patients by generating a patient‐specific virtual GRID block (software‐generated) and using IMRT technique to optimize the treatment plan. Here, we report our initial clinical experience using HT‐GRID, and dosimetric comparison results between HT‐GRID and LINAC‐GRID. This study evaluates 10 previously treated patients who had deep‐seated bulky tumors with complex geometries. Five of these patients were treated with HT‐GRID and replanned with LINAC‐GRID for comparison. Similarly, five other patients were treated with LINAC‐GRID and replanned with HT‐GRID for comparison. The prescription was set such that the maximum dose to the GTV is 20 Gy in a single fraction. Dosimetric parameters compared included: mean GTV dose ($D_{\text{GTV}}^{\text{mean}}$), GTV dose inhomogeneity (valley‐to‐peak dose ratio (VPR)), normal tissue doses ($D_{N}^{\text{mean}}$), and other organs‐at‐risk (OARs) doses. In addition, equivalent uniform doses (EUD) for both GTV and normal tissue were evaluated. In summary, HT‐GRID technique is patient‐specific, and allows adjustment of the GRID pattern to match different tumor sizes and shapes when they are deep‐seated and cannot be adequately treated with LINAC‐GRID. HT‐GRID delivers a higher $D_{\text{GTV}}^{\text{mean}}$, EUD, and VPR compared to LINAC‐GRID. HT‐GRID delivers a higher $D_{N}^{\text{mean}}$ and lower EUD for normal tissue compared to LINAC‐GRID. HT‐GRID plans also have more options for tumors with complex anatomical relationships between the GTV and the avoidance OARs (abutment or close proximity).

PACS numbers: 87.55.D, 87.55.de, 87.55.ne, 87.55.tg

## I. INTRODUCTION

Spatially fractionated radiotherapy (GRID) is generally used to treat patients using a conventional linear accelerator with either a commercially available GRID block or MLC‐based GRID pattern (LINAC‐GRID).^1^, ^2^, ^3^ It is used to treat large bulky malignant tumors and it has been shown to be an effective technique to enhance tumor local control.^4^, ^5^ However, LINAC‐GRID techniques face a major challenge to effectively treat bulky tumors with large skin to tumor distances. That is, there is inadequate dose to the target with most of the dose lost into normal tissue. Frequently, in such cases, the maximum dose ($D_{\text{max}}$) falls outside of the target. For helical tomotherapy, 51 projections or beams around the target are used to perform IMRT treatment planning and, because of the helical nature of the treatment delivery (the couch and gantry are moving during the treatment), it is possible for the system to deliver dose to deep‐seated targets. The University of Arkansas for Medical Sciences (UAMS) developed a customizable patient‐specific virtual GRID block to treat patients with the new TomoHDA V2.0 system (Accuracy Inc., Sunnyvale, CA) (HT‐GRID) to enable adequate GRID treatment of deep‐seated tumors. This is done by using a software‐generated customized virtual GRID block and an IMRT delivery technique. We have previously investigated the basic design of the virtual GRID block, the beam characteristics of HT‐GRID, and the possibility of applying HT‐GRID in the clinic.^(6)^ In the current study, we focused on the clinical application of HT‐GRID for deep‐seated bulky tumors (median distance from skin to the proximal edge of the tumor of 12.3 cm (range 3.4–26.6 cm)) and the dosimetric comparison between the HT‐GRID and LINAC‐GRID in a group of nonconsecutive patients treated at UAMS.

## II. MATERIALS AND METHODS

Ten previously treated patients who had deep‐seated bulky tumors with complex geometries were selected for this study. Among these patients, five patients were previously treated with HT‐GRID techniques and were replanned with LINAC‐GRID technique for comparison purposes. Another five patients were previously treated with LINAC‐GRID technique and were replanned with HT‐GRID technique for the comparison purposes as well. The prescription was set such that the maximum dose to the GTV is 20 Gy in a single fraction. Patient characteristics are presented in Table 1.

**Table 1:** Patient characteristics.

*Pt. #*	*Diagnosis*	*Target Location*	*Target Volume (cc)*	*Max. Target Dimension (cm)*	${\text{Pd}}_{\text{max}}$ ^a^ *(cm)*	${\text{Dd}}_{\text{max}}$ ^b^ *(cm)*
1	Renal cell carcinoma	Axilla	646	15.2	13.0	23.7
2	Squamous carcinoma of the head and neck of unknown primary	Neck	182	9.0	8.8	12.1
3	Renal cell carcinoma	Shoulder	293	9.0	5.1	9.5
4	Sarcoma	Lung	4646	27.9	12.6	32.3
5	Squamous carcinoma of the cervical esophagus	Neck	310	14.3	10.5	14.4
6	Squamous carcinoma of the larynx	Neck	269	13.9	12.8	14.5
7	Melanoma	Shoulder	391	14.6	3.4	15.2
8	Nonsmall cell lung cancer	Lung	1001	16.2	16.7	25
9	Squamous carcinoma	Anus	634	16.1	16.4	22.1
10	Carcinosarcoma	Uterus	4511	26.4	26.6	38.1

^a^ ${\text{Pd}}_{\text{max}}$: maximum distance from skin to proximal edge of the target.

^b^ ${\text{Dd}}_{\text{max}}$: maximum distance from skin to deepest part of target.

### A. LINAC‐GRID technique treatment plan

LINAC‐GRID treatment plans were generated using the Pinnacle v. 9.0 treatment planning station (TPS) (Philips Healthcare, Andover, MA) with a commercially available GRID block manufactured by Radiation Products Design, Inc. (Albertive, MN). Patient CT datasets were acquired on a CT‐simulator (Brilliance CT Big Bore, Philips, Cleveland, OH) and transferred to the Pinnacle TPS for contouring and planning. Patient GTV was contoured by the same physician. The normal tissue was defined as the volume of the planning CT dataset that received at least 5% of the prescription dose (100 cGy) as a region of very low dose minus the volume of GTV. The commercially available GRID block was imported to the Pinnacle TPS as a GRID block using the in‐house dedicated GRID script generated in Pinnacle TPS. LINAC‐GRID usually uses one beam angle or two beams in parallel opposed fashion.^(7)^ The details of LINAC‐GRID plans and their beam profiles were studied in our previous publications.^3^, ^6^

### B. HT‐GRID technique treatment plan

#### B.1 Virtual tomo GRID block

**Figure 1:**
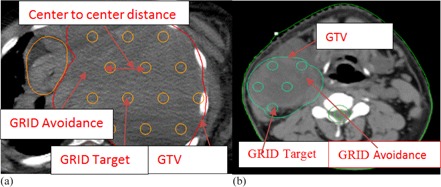
Illustration of HT‐GRID virtual GRID block (a) from Patient #4 (GTV volume=4646 cc); (b) virtual GRID block from Patient #1 (GTV volume=646 cc).

The virtual GRID block used in helical tomotherapy (HT) system were generated by in‐house software DICOMan.^(8)^ Two new structures (GRID Target and GRID Avoidance) were generated inside the GTV structure using DICOMan software. In summary, the software‐generated virtual GRID block consists of three elements: GTV (patient gross tumor volume), the GRID Target (the cylindrical structures or holes mimicking openings of commercially available GRID blocks inside the GTV), and the GRID Avoidance volume created by extracting the GRID Target from the GTV (mimicking the shielded area of the commercially available GRID block and thus used as an organ at risk, but still located inside the GTV). By changing the diameter of the opening holes and the center‐to‐center distance between the holes, DICOMan can configure the patient‐specific virtual GRID block in various settings of GRID Target and GRID avoidance structures (Figs. 1(a) and 1(b)).

#### B.2 HT‐GRID technique treatment plan

The same patient CT image datasets and same contours generated from Pinnacle TPS were transferred to the in‐house software DICOMan to generate the virtual GRID block. Patient CT and contours including the two new contours of GRID Target and GRID Avoidance were then transferred to the HT TPS for treatment planning. Figs. 1(a) and 1(b) show examples of the virtual GRID blocks. The Virtual GRID block is patient‐specific and can be optimized by manipulating the diameter of opening holes and the center‐to‐center distance between nearby holes in order to suit the particular patient GTV and OAR anatomical relationship. The virtual GRID block could be further modified after transferring to the HT TPS, to avoid hot or cold spots in the unintended areas.

**Figure 2:**
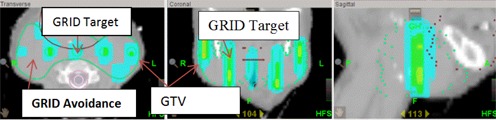
Illustration of HT‐GRID isodose distribution (transverse, coronal, sagittal plane) from Patient #6.

Prescribed dose was set to 20 Gy to the maximum point of the GTV in a single fraction. This is the same prescription as set in LINAC‐GRID. HT‐GRID used 6 MV X‐ray beams and IMRT treatment technique with dynamic jaw to treat the patients. The planning parameters such as field size, pitch number, and modulation factor need to be optimized to produce a treatment plan that is both dosimetrically acceptable and clinical deliverable. In HT, a long beam‐on time (BOT) is of concern because of potential patient motion. As such, optimization parameters were chosen that would reduce BOT. The 5 cm dynamic jaw, a pitch of 0.43, and a modulation factor of 1.5 were selected in this study to strike a fine balance between obtaining a dosimetrically acceptable and clinically deliverable plan while maximally reduce the BOT. For the HT optimization process, the GRID Target structure generated from DICOMan was used as the HT target constraint to gain a high dose inside the cylindrical structures of the virtual GRID block, while GRID Avoidance structure was set as the regions‐at‐risk constraints to limit dose as much as possible. This mimics the GRID beam delivery pattern. Figure 2 shows the example of the isodose distribution of the HT‐GRID.

From our previous experience of treating LINAC‐GRID based patients, the goal mean GTV dose was usually around 8 to 10 Gy,^3^, ^6^ whereas the goal for HT‐GRID treatment plan was to optimize the plan to reach a mean GTV dose of approximately 8–12 Gy. The difference in mean GTV dose between LINAC‐GRID and HT‐GRID takes into consideration the potential extension of the high‐dose region outside the GRID target. The dose to critical OAR was optimized by setting optimization goals to lower their doses. The systematic change of importance, dose objectives, dose‐volume objectives, and penalties were similar to our clinical experience in treating non‐GRID HT patients.^9^, ^10^

#### B.3 Dosimetric comparison between HT‐GRID and LINAC‐GRID treatment plans

The treatment plan evaluation parameters include equivalent uniform dose (EUD) and the mean dose ($D_{\text{GTV}}^{\text{mean}}$) for the GTV. The heterogeneity of the dose distribution inside the GTV for the patient was evaluated by the valley‐to‐peak dose ratio (VPR) and is defined as the ratio of minimum to maximum dose inside the GTV. Plan evaluation parameters for OARs include normal tissue mean dose ($D_{N}^{\text{mean}}$) and EUD, and the mean doses ($D_{\text{mean}}$) or maximum doses ($D_{\text{max}}$) for certain OARs.

The EUD is calculated as the following equation:^(11)^

(1) $$EUD={\left(\sum_{i=1}^{N}v_{i}D_{i}^{a}\right)}^{\frac{1}{a}}$$

In the equation above, *a* is a unitless model parameter describing the dose response of each structure and $v_{i}$ is the weight of the ith partial volume receiving dose $D_{i}$ in Gy. This model can be used for both tumors and normal tissue. In this study, we use a=−5 for tumor and a=5 for normal tissue. These values were selected based on our prior experience with IMRT treatment planning using the EUD as a constraint/objective. However, the value of EUD with a large negative value of “a” is close to the minimal dose which is a characteristic consistent with the response of highly inhomogeneously irradiated tumors. On the other hand, for a large positive value of the parameter “a”, the EUD is near the maximum dose. This is consistent with nonuniformly irradiated serial normal structures.^(12)^

Tomotherapy treatment plan can export differential DVH (including corresponding partial volume $v_{i}$ or ith partial volume) to an excel file. Differential EUD was calculated in the excel file. The total EUD was then calculated by adding each differential EUD.

#### B.4 HT‐GRID treatment plan dose verification

Patient‐specific quality assurance (QA) for HT‐GRID treatment plan was performed using a PTW OCTAVIUS phantom and 729 2D ion chamber array (PTW, Freiburg, Germany). The dose measurement results were analyzed using the PTW‐VeriSoft software.

## III. RESULTS

Deep‐seated bulky tumors were selected in this study. The median tumor volume was 634 cc (182–4646 cc). The median distance from skin to the proximal edge of the tumors was 12.7 cm (range 3.4–26.6 cm) and the median distance from skin to the deepest part of tumor was 22 cm (range 8.9–38 cm).

The results for GTV EUD, ($D_{\text{GTV}}^{\text{mean}}$) and VPR and the results for normal tissue $D_{N}^{\text{mean}}$ and EUD, and results of OAR doses from both HT‐GRID and LINAC‐GRID treatment plans for the selected 10 patients are listed in Tables 2, 3 and 4.

The median mean GTV dose was 6.73 Gy (4.45–8.50 Gy) for LINAC‐GRID, and was 10.65 Gy (9.83‐12.59 Gy) for HT‐GRID. The median GTV EUD for LINAC‐GRID treatment plan was 3.95 Gy (0.15‐4.73 Gy), and was 7.62 Gy (4.31‐11.06 Gy) for HT‐GRID treatment plan.

The median normal tissue mean dose was 1.24 Gy (0.34–2.54 Gy) for HT‐GRID and 0.61 Gy (0.11–1.52 Gy) for LINAC‐GRID; the median normal tissue EUD for HT‐GRID was 5.45 Gy (3.45–6.89 Gy) and 6 Gy (4.45–6.82 Gy) for LINAC‐GRID plans.

**Table 2:** GTV EUD, $D_{\text{GTV}}^{\text{mean}}$ and VPR for HT‐GRID and LINAC‐GRID.

	*EUD (cGy)*		$D_{\text{GTV}}^{\text{mean}}$ *(cGy)*		*VPR*	
*Pt.#*	*HT*	*LINAC*	*HT*	*LINAC*	*HT*	*LINAC*
1	431	403	1023	612	0.064	0.07
2	807	473	1108	850	0.18	0.12
3	753	388	1102	847	0.14	0.054
4	753	410	1020	623	0.18	0.14
5	753	15	1043	850	0.15	0.079
6	1106	420	1259	799	0.29	0.056
7	860	285	1085	723	0.20	0.024
8	771	24	1045	596	0.14	0.002
9	448	408	983	613	0.05	0.0001
10	925	295	1105	445	0.07	0.0008

**Table 3:** Normal tissue $D_{N}^{\text{mean}}$ and EUD for HT‐GRID and LINAC‐GRID.

*Dose (cGy)*	$D_{\text{mean}}^{N}$	*EUD*	$D_{\text{mean}}^{N}$	*EUD*	$D_{\text{mean}}^{N}$	*EUD*	$D_{\text{mean}}^{N}$	*EUD*	$D_{\text{mean}}^{N}$	*EUD*
*Pt. #*	*1*		*2*		*3*		*4*		*5*	
HT	145	569	92	572	120	656	207	345	34	345
LINAC	81	593	60	608	62	629	132	682	20	445
Diff^a^	64	−24	64	−36	58	27	119	−337	14	−100
*Pt. #*	*6*		*7*		*8*		*9*		*10*	
HT60	522	125	474	124	598	160	371	254	689	
LINAC	21	474	45	573	11	641	152	667	96	513
Diff^a^	39	47	80	−99	113	−43	8	−295	158	176

^a^ Diff=the dose difference between HT‐GRID and LINAC‐GRID (cGy).

**Table 4:** OAR doses for 10 selected patients from HT‐GRID and LINAC‐GRID.

*Dose (cGy)*	*HT*	*LINAC*	*HT*	*LINAC*	*HT*	*LINAC*	*HT*	*LINAC*	*HT*	*LINAC*
*Pt. #*	*1*		*2*		*3*		*4*		*5*	
Cord^a^	214	716	174	966	166	4	445	968	272	69
Lt. Humerus^b^	343	29	–	–	–	–	–	–	–	–
Rt. Parotid^a^	–	–	707	251	–	–	–	–	–	–
Oral cavity^a^	–	–	360	1673	–	–	–	–	–	–
Lt. Plexus^a^	–	–	–	–	374	309	–	–	–	–
Rt. Plexus^a^	–	–	–	–	–	–	283	402	–	–
Larynx	–	–	–	–	–	–	–	–	1258	1028
*Pt. #*	*6*		*7*		*8*		*9*		*10*	
Dose (cGy)	HT	Linac	HT	Linac	HT	Linac	HT	Linac	HT	Linac
Cord^a^	355	1577	209	12	612	1363	–	–	–	–
Oral cavity^a^	1746	1877	–	–	–	–	–	–	–	–
Rt. Lung^b^	–	–	276	95	–	–	–	–	–	–
Esophagus^b^	–	–	–	–	353	281	–	–	–	–
Bowel^a^	–	–	–	–	–	–	959	1041.0	1219	1147

^a^ $D_{\text{max}}$.

^b^ Dmean.

−=OAR not of interest for this patient.

**Figure 3:**
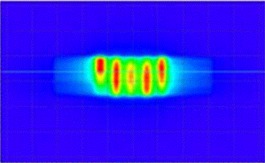
Patient QA dose map (coronal plane) measurement result for HT‐GRID technique using PTW OCTAVIUS phantom and 729 2D ion chamber array from Patient #6.

HT‐GRID patient QAs were performed and the results showed that the gamma agreement index score was 97.8% and higher (3%, 3 mm). Figure 3 shows the measurement dose map result for coronal plane from Patient #6. The QA result shows that radiation high dose was only delivered inside the cylindrical structure of the GRID Target within GTV and it had a good agreement compared with the calculated dose map (coronal plane) from same patient showed in Fig. 2.

## IV. DISCUSSION

HT‐GRID technique with the novel virtual GRID block was successfully applied in our clinic. In this paper, deep‐seated bulky tumors especially with large distances from skin to tumor were selected. Treatment plans and dosimetric parameters were compared between HT‐GRID and LINAC‐GRID treatment plans and several characters for HT‐GRID treatment plan are discussed.

The mean GTV doses were compared between HT‐GRID and LINAC‐GRID plans and the comparison results showed that the mean GTV doses from HT‐GRID were generally higher than the LINAC‐GRID treatment plan. This is mainly because the HT‐GRID plan had larger VPR compared to the LINAC‐GRID plan (median VPR was 0.145 with a range of 0.05 to 0.29 for HT‐GRID and was 0.055 with a range of 0.0001 to 0.14 for LINAC‐GRID). Compared to the commercially available LINAC block, the virtual GRID block is not a real physical block. The characteristics of the virtual GRID block and the nature of HT beams can result in the extension of the high‐dose region from the open area (GRID Target) to the block area (GRID avoidance) within the GTV.

**Figure 4:**
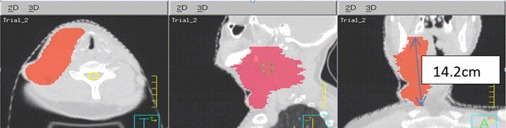
Illustration of irregular deep‐seated bulky tumor from Patient #5.

**Figure 5:**
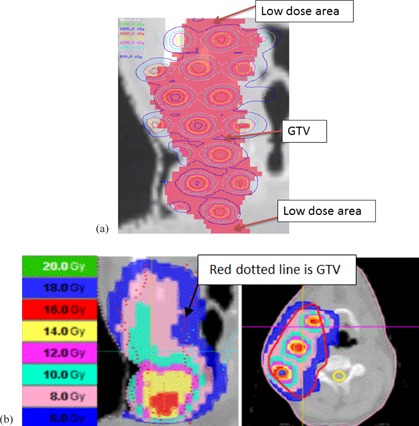
Isodose distribution (a) from LINAC‐GRID Patient # 5 (illustration of low‐dose area in superior and inferior portions of the tumor volume in coronal plane); isodose distribution (b) from HT‐GRID Patient #5 (coronal plan from same CT slice as compared to LINAC‐GRID and transverse plane).

We did not find any GTV EUD studies in the literature. The comparison results showed that the GTV EUDs were substantially higher for HT‐GRID treatment plans compared to the EUDs from LINAC‐GRID treatment plans. This study also found that, for some patients with LINAC‐GRID treatment plan, the GTV EUDs were very low even though the mean doses were within the 7–12 Gy range. This was due to portions of very low dose inside the GTV. For example, Patient #5, the EUD was only 0.15 Gy, even though the mean GTV dose was 8.5 Gy. For the same patient with an HT‐GRID treatment plan, the EUD was 7.53 Gy and the mean GTV dose was 10.43 Gy. For this specific patient, the shape of the GTV was very irregular (maximum dimension was 14.2 cm in the superior and inferior dimension, and the tumor was 2.4 times wider in the sagittal plane as compared to the coronal plane) (Fig. 4). Because of the elongated GTV shape along the superior and inferior direction, one single GRID field could not cover the entire GTV volume when using LINAC‐GRID; in addition, this patient had a head and neck cancer where many critical structures were in the vicinity of the GTV. As a result, there were relatively larger portions of low‐dose volumes near the superior and inferior end of the GTV (Fig. 5(a)). By definition, the EUD is calculated by finding the sum of all partial volumes receiving dose in the GTV structure. Hence, any partial volumes receiving a dose close or near zero would yield a very low GTV EUD.

For HT‐GRID, the virtual GRID block can be modified by adding more open holes in the low dose area at the superior and inferior direction of the GTV to improve on the low‐dose area. In addition by using IMRT technique, GTV EUD can be improved upon, while maintaining the GRID beam characteristics and sparing the critical OARs. It is important to note that varying the diameter of the hole and the center‐to‐center spacing can affect the therapeutic ratio.^(13)^ However, it is beyond the scope of this paper to determine the magnitude of this change.

Figure 5(b) shows the dose distribution at coronal plane for the same CT slice cut as compared to LINAC‐GRID plan. The 600 cGy dose distribution (blue color) covered more of the GTV compared to LINAC‐GRID plan and no large cold spot area at the superior and inferior direction of the GTV was seen.

**Figure 6:**
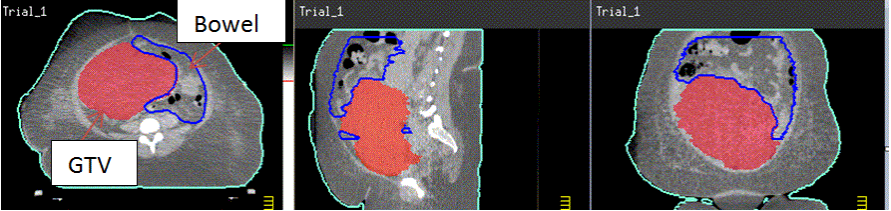
Illustration of large volume of GTV with critical organ (small bowel) around it from Patient #10.

Another example is Patient #10 which had a very large volume GTV (4511 cc) and a critical organ of small bowel abutting the GTV (Fig. 6). The GTV EUD was 2.95 Gy for LINAC‐GRID plan and 9.25 Gy for the HT‐GRID plan for the same reasons.

**Figure 7:**
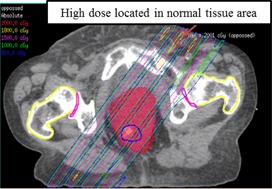
Isodose distribution for LINAC‐GRID plan using parallel opposed beams from Patient #9.

The literature provides data regarding the use of parallel opposed GRID therapy to improve upon the dose distribution of the GRID GTV when treating deep‐seated tumors. Meigooni et al.^(7)^ reported on GRID therapy using parallel opposed beams. It was shown that parallel opposed GRID is a viable option for treatment of deep‐seated tumors as the spatially fractionated dose distribution is preserved. However, this approach can expose the volume of normal tissue proximal and distal to the target to potentially high doses of radiation (Fig. 7). This is mainly due to the deep‐seated nature of the large tumors. Table 1 shows the range of the maximum distance from the skin to the proximal edge of the target was from 3.4 cm to 22.6 cm for the patients selected in our study. So one or two radiation beams (adjacent but nonopposing fields) were used in this study, depending on the tumor volume and location, to reduce normal tissue exposure.

Normal tissue mean doses were generally higher for HT‐GRID plan compared to LINAC‐GRID plan. This result can be explained by the following reasons. HT‐GRID plans used an IMRT technique, whereas only one or two static field beams were delivered for LINAC‐GRID plan. In addition, the HT beam is delivered in a helical fashion, so the integral dose is usually higher compared to LINAC radiotherapy using static beams. Our study results showed the normal tissue mean dose was higher for HT‐GRID plan compared to LINAC‐GRID plans and that result was in agreement with the reported results from other published papers.^(14)^

**Figure 8:**
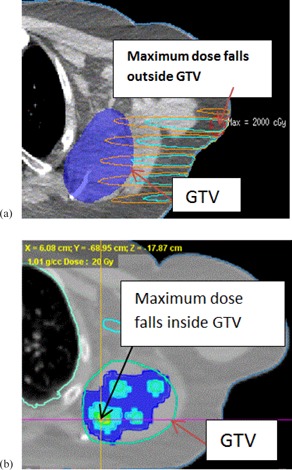
LINAC‐GRID plan (a) in which maximum dose falls outside the GTV, from Patient #1; HT‐GRID plan (b) where maximum dose (cross line) falls inside the GTV, from Patient #1.

However, HT‐GRID generally has lower normal tissue EUD compared to LINAC‐GRID plans. This could be explained by several reasons. The LINAC‐GRID plan only used one or two static beams without any beam modulation, for the deep‐seated tumors selected in this study, and the maximum doses all fell outside the GTV target (Figs. 8(a) and 8(b)). However, by using IMRT technique in HT‐GRID plans, there is more control of the dose deposition within the GTV and the maximum dose could be confined within the GTV. In this way, the plan could be optimized by adding different constraints to GTV target and OARs. In addition, planning structures, such as rings, can be used to further avoid the high dose outside the GTV target. In this study, the maximum doses all fell inside the GTV target. In results, HT‐GRID plans had lower normal tissue dose near the edge of the GTV and higher normal tissue dose beyond the GTV boundary compared to LINAC‐GRID, so the EUD over the entire normal tissue was lower compared to LINAC‐GRID plans.

However, there was an exception for Patient #10, where the HT‐GRID plans had a higher than normal tissue EUD, compared to the LINAC‐GRID plan (689 cGy vs. 513 cGy). This patient had a very large, deeply seated GTV (4511 cc) (Fig. 7), and a portion of the GTV that received near zero doses in the LINAC‐GRID plan minimized the EUD results of the LINAC‐GRID plan compared to the HT‐GRID plan. For HT‐GRID, in order to constrain the bowel dose, the HT beams were highly intensified and, as a result, increased the normal tissue EUD.

**Figure 9:**
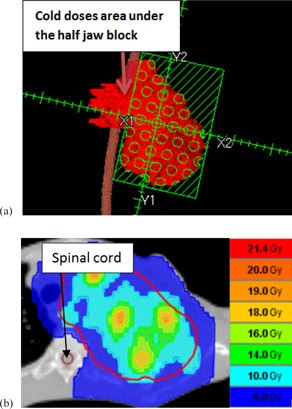
LINAC‐GRID (a) with half jaw block to spare the cord dose (Patient #8); HT‐GRID (b) with sparing cord dose by modifying the HT virtual GRID block and using IMRT technique (Patient #8).

OARs doses were also compared in this study. Generally the OARs doses were comparable between the HT‐GRID plan and the LINAC‐GRID plan. However, occasionally it is very difficult to avoid the beam passing through a specific OAR in LINAC‐GRID plans. For example, in the case of Patient #8, the spinal cord could be blocked by closing half jaw (Fig. 9(a)) to reduce the spinal cord dose; however, this would underdose a substantial portion of the GTV and, as a result, reduce its EUD.

For the same patient, if the spinal cord is not excluded from the beam's path, the maximum spinal cord dose would be 13.62 Gy for a single fraction. This would carry an unacceptable risk of spinal cord myelitis. HT‐GRID could solve this problem by modifying the virtual GRID block to both maximize exclusion of the spinal cord and minimize the portion of the GTV not receiving any dose. Additionally, the use of inverse planning in HT‐GRID allows for optimization of dose deposition within the GTV (Fig. 9(b)). This study showed that HT‐GRID plan could substantially reduce the spinal cord dose while keeping the comparable or better GTV dose distribution, compared to the LINAC‐GRID plan (Table 4).

Another example is Patient #10 (Fig. 7) who had a large GTV (4511 cc) and a small bowel structure abutting it. For LINAC‐GRID, a single, large, static field would not be enough to cover the entire GTV and multiple beams would be needed to receive the acceptable GTV dose. However, this would significantly increase the small bowel dose to an unacceptable level. An HT‐GRID plan could reduce the bowel dose by closing some open areas of the GTV near the small bowel structure and, together with IMRT optimization, reach the acceptable GTV dose and spare the small bowel dose to an acceptable level.

## V. CONCLUSIONS

Patients who are eligible to receive GRID therapy were successfully treated using HT‐GRID treatment technique at UAMS. By generating the virtual GRID block, HT‐GRID can mimic the dose delivery pattern defined by LINAC‐GRID treatments with a commercially available GRID block. The virtual GRID block needs to be optimized for each individual patient in order to get best GTV dose coverage while sparing the OAR doses to the acceptable level. In this study, both HT‐GRID and LINAC‐GRID treatment techniques have their pros and cons. The LINAC‐GRID had smaller VPR and was easy to plan, whereas HT‐GRID planning is better suited for GTVs showing large skin‐to‐tumor distance or complex anatomical relationship between the GTV and the OAR (abutment or close proximity). LINAC‐GRID has limitations in treating this type of deep‐seated tumor as seen in Patients #5 and #8 who were not suited for LINAC‐GRID treatment. This stresses the importance of EUD in these cases, as the average doses to the GRID GTV seemed appropriate but the EUD was very low — thus indicating that the treatment may be a failure. In addition, HT‐GRID uses IMRT optimization to enhance the dose distribution on the GTV. EUD, in conjunction with the mean GTV dose, was found to be a good indicator to evaluate the GRID dose coverage.
